# Case report: Abrocitinib: a potential therapeutic option for lichen amyloidosis associated with atopic dermatitis

**DOI:** 10.3389/fimmu.2024.1477664

**Published:** 2024-11-14

**Authors:** Yizhen Zhang, Dawei Huang, Yunlu Gao

**Affiliations:** ^1^ School of Medicine, Tongji University, Shanghai, China; ^2^ Department of Dermatology, Shanghai Skin Disease Hospital, Shanghai, China

**Keywords:** abrocitinib, atopic dermatitis, lichen amyloidosis, small molecule drugs, case report

## Abstract

Lichen amyloidosis (LA) is a predominant type of primary cutaneous amyloidosis that is characterized by persistent and intense skin itching. Although multiple therapeutics strategies are available for its treatment, there is no standard treatment so far. Abrocitinib, an oral small-molecule Janus kinase 1 inhibitor, has been authorized for the treatment of severe atopic dermatitis (AD) and can also provide rapid relief from pruritus. Here, we discuss the case of a 32-year-old man who was diagnosed with LA with severe AD based on the presence of multiple, discrete, and hyperpigmented papules and pruritic, erythematous macules with lichenification of the limbs, trunk, and buttocks. Given the inefficacy of conventional therapy, abrocitinib treatment was recommended in this patient. After 1 month of treatment, the patient’s Eczema Area And Severity Index score decreased significantly from 48 to 15 points, accompanied by a notable reduction in pruritus symptoms. Furthermore, significant improvements were observed in the thickness and pigmentation of the hyperkeratotic papules. Thus, abrocitinib exhibited excellent effectiveness and safety in the treatment of severe AD with LA and warrants further investigation for its potential therapeutic benefits.

## 1 Introduction

Lichen amyloidosis (LA) is a form of primary cutaneous amyloidosis that is characterized by numerous, itchy, hyperpigmented, and hyperkeratinized papules that appear on the anterior shins, lateral arms, and lower back (1). LA is a feature of a variety of skin diseases that eventually lead to pruritus, including atopic dermatitis (AD) (2). Although several therapeutic approaches have been proposed for the treatment of LA, such as topical and intralesional corticosteroids, oral antihistamines, systemic cyclosporine, and UV radiation, none of them have demonstrated consistency in their effectiveness (2–4).

Abrocitinib, a small-molecule inhibitor of Janus kinase 1 (JAK1), modulates multiple cytokine pathways involved in several inflammatory cutaneous diseases. It is authorized for the treatment of moderate to severe AD and also provides rapid relief from pruritus (5, 6). Because LA might be related to the onset of AD, small-molecule drugs for AD are currently being used as novel therapeutic agents for the treatment of LA with promising results. Here, we describe a case of LA associated with severe AD in which the patient responded well to treatment with abrocitinib after conventional therapy was ineffective.

## 2 Case report

**Figure 1:**
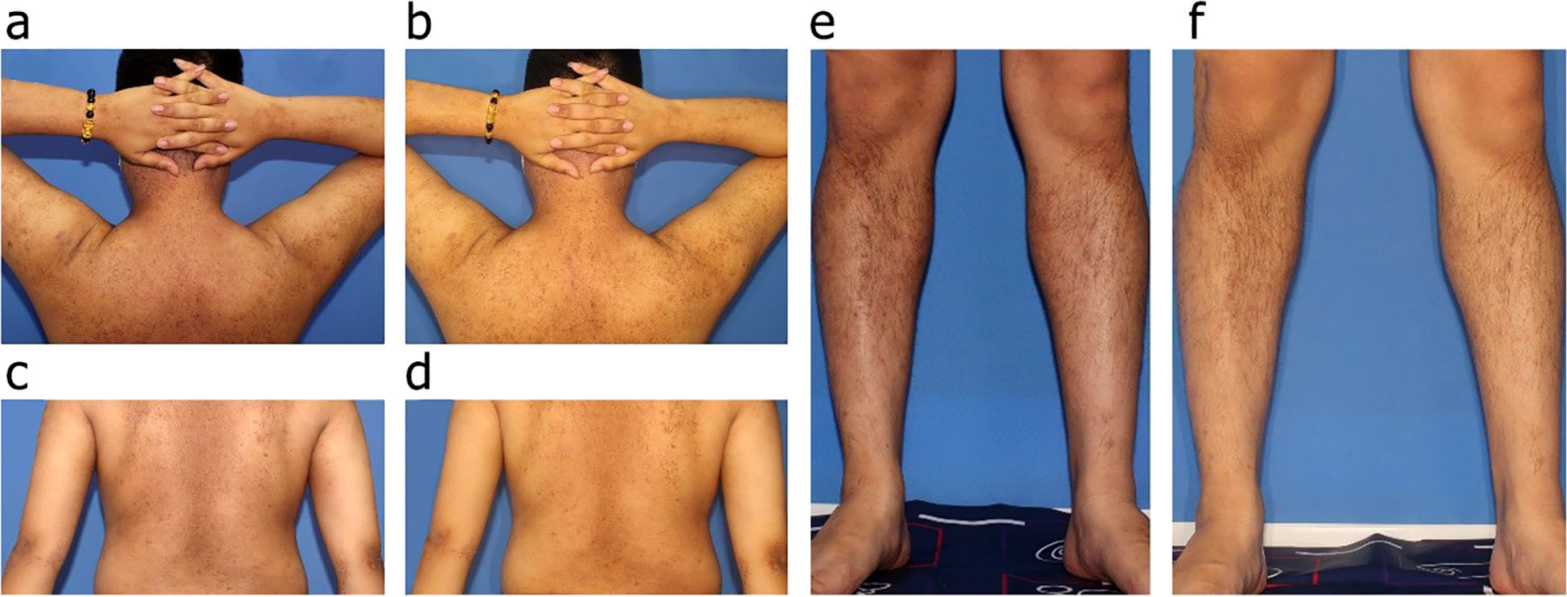
**(A, C, E)** Clinical images of the patient with LA associate with AD before abrocitinib treatment, illustrating multiple, discrete, firm, and hyperpigmented papules present on the extensor surfaces of the limbs, trunk, and buttocks. **(B, D, F)** Clinical images of the patient 4 weeks after treatment with abrocitinib, demonstrating that all papules had flattened and exhibited a reduction in both size and number.

**Figure 2:**
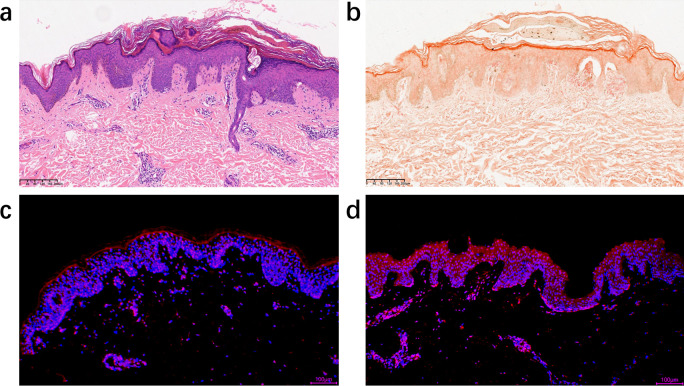
**(A)** Histopathological changes consistent with LA, uniform eosinophilic substance in the papillary dermis that covered the hyperplastic epidermis (hematoxylin-eosin; 10×). **(B)** A substantial accumulation of fibrous amyloid deposits is observed beneath the dermal-epidermal junction, which stain orange with Congo red. Notably, distinct fissures are present between these deposits (10×). **(C)** Immunofluorescence staining of IL-31 expression in a pathological section of normal skin (10×). IL-31 were stained in red, whereas nuclei were stained in blue 4',6-diamidino-2-phenylindole (DAPI). **(D)** Immunofluorescence staining of IL-31 expression in a pathological section of the patient in this case. IL-31 infiltrated in the epidermis of the patient and expressed at significantly higher levels compared to that in normal human skin tissues.

A 32-year-old man with a 20-year history of refractory AD presented to our department with severe, widespread uncontrollable pruritus. Physical examination showed typical chronic eczema-like changes in the form of numerous, discrete, and hyperpigmented papules on the back, neck, and lower legs that were particularly prominent on the lower legs (**Figures 1A, C, E**). Dryness, scaling, pigmentation, and irregularly shaped lichenoid plaques due to repeated scratching were observed on the neck and upper back, areas that are easily accessible to the patient (**Figure 1A**). A punch biopsy of the right arm revealed the presence of a uniform eosinophilic substance in the papillary dermis that covered the hyperplastic epidermis, and Congo red staining demonstrated a substantial accumulation of fibrous amyloid deposits located beneath the dermal-epidermal junction, representing a characteristic manifestation of LA (**Figures 2A, B**). Additionally, immunofluorescence analysis indicated that the expression of Interleukin-31 (IL-31) in the skin of this patient was significantly elevated compared to that in a pathological section of normal skin (**Figures 2C, D**). Microscopic examination of the underlying causes of itching, including scabies, yielded negative results. According to the history and typical clinical features, the patient was diagnosed with LA with severe AD [Eczema Area and Severity Index (EASI) = 48]. Topical corticosteroids and antihistamines were not considered because they had previously failed to alleviate patient’s discernible clinical manifestations of AD and LA. In addition, the use of duplizumab was contraindicated because of the patient’s history of allergic conjunctivitis. Therefore, treatment with abrocitinib was recommended. The patient was administered 100 mg of abrocitinib orally once daily, which resulted in alleviation of intractable pruritus within 2 days of treatment. Within 2 weeks, the patient experienced relief from unbearable itching, and the severity of pruritus decreased from 10 points on the Numeric Rating Scale to only 1 point. After 1 month of treatment, the patient’s EASI score significantly decreased from 48 to 15. This was accompanied by a decrease in the size and number of plaques on the back and lower legs, significant flattening of the LA papules, and gradual easing of the desquamation (**Figures 1B, D, F**). So far, this patient has been on continuous 3-month course of abrocitinib with no recurrence of pruritus and sustained clinical benefit. The patient has not exhibited any of the known side effects of abrocitinib, such as infection, transaminase abnormalities, cardiotoxicity, and skeletal dysplasia.

## 3 Discussion

LA is a chronic pruritic type of cutaneous amyloidosis that results from extracellular deposition of amyloid-derived intermediate filaments in the dermis, and the itch-scratch cycle plays a crucial role in its pathogenesis. Although various conventional treatments, such as topical corticosteroids, oral antihistamines, and systemic cyclosporine, have been used for LA with AD, these treatments are frequently unsatisfactory or provide relief from the itching without regression of the lesion (7). Recently, successful treatment of a few cases of LA has been reported with small-molecule drugs and biological agents such as dupilumab, upadacitinib, and baricitinib (8–10). For example, Dengmei et al. (10) reported that, in a patient with refractory LA coexisting with AD, the LA lesions significantly improved and eczema-like lesions and pruritus rapidly resolved after 16 weeks of baricitinib treatment. Further, Qingzhu et al. (11) reported four cases of refractory LA with AD that were treated with dupilumab after conventional therapy had proved ineffective. After 16 weeks of treatment with the biologic agent, reduction in the number of skin lesions and improved quality of life were observed in all the patients, with no significant adverse effects. In contrast to the previous case, in our case, the use of the biological agent dupilumab was contraindicated because of the patient’s history of allergic conjunctivitis. Therefore, abrocitinib, a small-molecule drug also approved for the treatment of AD, was used in this case, with excellent therapeutic results.

Abrocitinib, a selective JAK1 inhibitor, affects epidermal barrier modulation and peripheral nerve modulation involved in pruritus transduction by targeting the JAK- signal transducer and activator of transcription (STAT) signaling pathway (12). Although the pathogenesis of LA is unclear, IL-31, one of the cytokines involved in the JAK-STAT signaling pathway, is considered to be a central mediator of T-cell–mediated pruritus. Furthermore, several articles have indicated that pruritus in LA may be correlated with the hypersensitivity of dermal nerve fibers, which are linked to IL-31 receptors in the epidermis (13). JAK1 inhibitors are known to directly affect T-cell function; moreover and importantly, blockade of IL-31 and IL-4 signaling by these inhibitors in primary afferent sensory neurons can influence inflammation and neurosensory pathways (14, 15). In our case, significant increases in the expression of IL-31 associated with pruritus were observed in the patient’s skin, with pruritus being observed in the patient’s skin, with rapid relief of itching following treatment with abrocitinib. Thus, the mechanism of abrocitinib in LA may involve reduction in the levels of the inflammatory cytokine IL-31 and repair of the damaged skin barrier.

The observations of this case report indicate that abrocitinib could be a promising therapeutic agent for the treatment of LA in patients with AD. However, more cases are required to assess the long-term efficacy of small-molecule drugs and their sustained effects after withdrawal. In addition, future studies need to focus on the mechanism of abrocitinib in relieving the pruritus associated with LA, as this may provide deeper insight into LA therapy.

## Data Availability

The original contributions presented in the study are included in the article/supplementary material. Further inquiries can be directed to the corresponding author.

## References

[1] Hamie L Haddad I Nasser N Kurban M Abbas O. Primary localized cutaneous amyloidosis of keratinocyte origin: an update with emphasis on atypical clinical variants. Am J Clin Dermatol. (2021) 22:667–80. doi: 10.1007/s40257-021-00620-9 34286474

[2] Behr FD Levine N Bangert J. Lichen amyloidosis associated with atopic dermatitis: clinical resolution with cyclosporine. Arch Dermatol. (2001) 137:553–5. doi: 10-1001/pubs 11346332

[3] Carlesimo M Narcisi A Orsini D Mari E Di Russo P Arcese A. A case of lichen amyloidosus treated with acitretin. Clin Ter. (2011) 162:e59–61. 21533310

[4] Shimoda Y Sato Y Hayashida Y Yamazaki Y Mizukawa Y Nakajima K. Lichen amyloidosus as a sweat gland/duct-related disorder: resolution associated with restoration of sweating disturbance. Br J Dermatol. (2017) 176:1308–15. doi: 10.1111/bjd.15060 27628905

[5] Deeks ED Duggan S. Abrocitinib: first approval. Drugs. (2021) 81:2149–57. doi: 10.1007/s40265-021-01638-3 PMC8917037 34807428

[6] Eichenfield LF Flohr C Sidbury R Siegfried E Szalai Z Galus R. Feeney C et al: Efficacy and Safety of Abrocitinib in Combination With Topical Therapy in Adolescents With Moderate-to-Severe Atopic Dermatitis: The JADE TEEN Randomized Clinical Trial. JAMA Dermatol. (2021) 157:1165–73. doi: 10.1001/jamadermatol.2021.2830 PMC8374743 34406366

[7] Weidner T Illing T Elsner P. Primary localized cutaneous amyloidosis: A systematic treatment review. Am J Clin Dermatol. (2017) 18:629–42. doi: 10.1007/s40257-017-0278-9 28342017

[8] Aoki K Ohyama M Mizukawa Y. A case of lichen amyloidosis associated with atopic dermatitis successfully treated with dupilumab: A case report and literature review. Dermatol Ther. (2021) 34:e15005. doi: 10.1111/dth.15005 34037298

[9] Solimani F Dilling A Ghoreschi FC Nast A Ghoreschi K Meier K. Upadacitinib for treatment- resistant Lichen amyloidosis. J Eur Acad Dermatol Venereol. (2023) 37:e633–5. doi: 10.1111/jdv.18756 36394121

[10] Xia D Xiao Y Li M Li W. Refractory cutaneous lichen amyloidosis coexisting with atopic dermatitis responds to the Janus Kinase inhibitor baricitinib. Dermatol Ther. (2022) 35:e15724. doi: 10.1111/dth.15724 35855568

[11] Zhu Q Gao BQ Zhang JF Shi LP Zhang GQ. Successful treatment of lichen amyloidosis coexisting with atopic dermatitis by dupilumab: Four case reports. World J Clin cases. (2023) 11:2549–58. doi: 10.12998/wjcc.v11.i11.2549 PMC10131001 37123319

[12] Huang IH Chung WH Wu PC Chen CB. JAK-STAT signaling pathway in the pathogenesis of atopic dermatitis: An updated review. Front Immunol. (2022) 13:1068260. doi: 10.3389/fimmu.2022.1068260 36569854 PMC9773077

[13] Tey HL Cao T Nattkemper LA Tan VW Pramono ZA Yosipovitch G. Pathophysiology of pruritus in primary localized cutaneous amyloidosis. Br J Dermatol. (2016) 174:1345–50. doi: 10.1111/bjd.2016.174.issue-6 26748444

[14] Cevikbas F Wang X Akiyama T Kempkes C Savinko T Antal A. Buddenkotte J et al: A sensory neuron-expressed IL-31 receptor mediates T helper cell- dependent itch: Involvement of TRPV1 and TRPA1. J Allergy Clin Immunol. (2014) 133:448–460. doi: 10.1016/j.jaci.2013.10.048 24373353 PMC3960328

[15] Wohlrab J Stintzing D Schultz L Jugelt K Schroeder OH. Influence of janus kinase inhibitors on the neuronal activity as a proof-of-concept model for itch. Skin Pharmacol Physiol. (2022) 35:94–101. doi: 10.1159/000519669 34530431 PMC8985001

